# The Importance of Subchondral Bone in the Pathophysiology of Osteoarthritis

**DOI:** 10.3389/fvets.2018.00178

**Published:** 2018-08-28

**Authors:** Holly L. Stewart, Christopher E. Kawcak

**Affiliations:** Equine Orthopaedic Research Center, Department of Clinical Sciences, College of Veterinary Medicine and Biomedical Sciences, Colorado State University, Fort Collins, CO, United States

**Keywords:** subchondral bone, osteochondral unit, repetitive stress, chronic fatigue, exercise, horse, osteoarthritis

## Abstract

Subchondral bone plays a critical role in the pathogenesis of osteochondral disease across veterinary species. The subchondral bone is highly adaptable, with the ability to model and remodel in response to loading stresses experienced by the joint. Repetitive stress injuries within the joint can result in primary or secondary pathologic lesions within the subchondral bone, which have been recognized to contribute to the development and progression of osteoarthritis. Recent advances in diagnostic imaging, particularly volumetric imaging modalities have facilitated earlier identification of subchondral bone disease. Despite these advancements, limitations in our knowledge about subchondral bone makes treatment and prevention of these conditions challenging. The purpose of this report is to review our current understanding of subchondral bone and its relationship to osteoarthritis across veterinary species, with a specific focus in the research that has been performed in horses. It can be concluded that our current understanding of subchondral bone is advancing, and future experimental, clinical and pathologic studies will provide additional insight about subchondral bone and its relationship to joint disease.

## Introduction

As our understanding of the underlying pathophysiology of osteoarthritis (OA) grows, we have begun to recognize that OA is a disease of not just articular cartilage, but of the osteochondral unit. The osteochondral unit is composed of articular cartilage, calcified cartilage, and subchondral and trabecular bone, which work synergistically to support functional loading of the joint (1, 2). Subchondral bone has received particular attention in recent years, as derangements in this essential tissue have been recognized for its contribution to the development and progression of OA. In this review, we will begin with a brief discussion of the anatomy, physiology, and biomechanical principles that guide subchondral bone function. We will then delve into the specific conditions of subchondral bone, specifically subchondral bone disease, repetitive stress injury, and chronic fatigue injury. We will conclude with some general principles of diagnosis and a brief discussion of treatment and prevention strategies.

## Anatomy and physiology of subchondral bone

The subchondral bone is located deep to the articular cartilage, but remains connected to it through a layer of calcified cartilage. The subchondral bone varies in architecture and physiology by region, from the more compact layer of bone adjacent to the calcified cartilage (subchondral bone plate), to the trabecular bone closer to the medullary cavity. Normal subchondral bone plate is a thin layer of bone ranging from 10 μm to 3 mm in thickness, depending on the location (3). The character of subchondral bone also differs depending on the thickness: the thinner areas are predominantly appositional layers continuous with trabeculae and with a low number of haversian canals; while the thicker areas are composed predominantly of a network of osteons (4). The function of the subchondral bone is to attenuate forces generated through locomotion, with the compact subchondral bone plate providing firm support and the subchondral trabecular component providing elasticity for shock absorption during joint loading (3). Maintenance of this intrinsic joint elasticity is essential for the biomechanical principles of locomotion.

Subchondral bone is a biphasic material, which includes an inorganic component composed of hydroxyapatite crystals for rigidity, and an organic component composed of predominantly type I collagen, proteoglycan, glycosaminoglycans, and water affording elasticity and pliancy (3). The composition of subchondral bone is uniquely designed to disperse axial loads across the joint, sparing the overlying articular cartilage (5–8). Subchondral bone has the innate ability to display a range of responses, reflecting both acute stresses as well as more prolonged, chronic, adaptive responses within the joint. At one end of the spectrum, subchondral bone is responsible for dissipating forces generated by locomotion, considering it has been shown to be 10 times more deformable than the cortical shaft of long bones (9). Further along the spectrum, subchondral bone is able to physically adapt its morphology in response to stresses placed on the joint. The adaptive capabilities of subchondral bone follow Wolff's Law, which states that bone will adapt in response to the loading under which it is subjected (10). The adaptive response is facilitated through the formative and resorptive activities of osteoblasts and osteoclasts, respectively, and is macroscopically observed within the trabecular bone. The rich vascularization and innervation of subchondral bone facilitates a comprehensive and extensive local response to both physiologic and pathologic alterations within the bone.

## Biomechanics and pathophysiology of subchondral bone

Unlike other tissues within the joint, subchondral bone is highly responsive to loading, with the ability to respond quickly to training and injury (11). The forces incurred by the articular cartilage are transmitted to the subchondral bone across the calcified cartilage layer, which is uniquely adapted to distribute forces and minimize shear stresses on the articular cartilage layer through an undulating association with subchondral bone (12). Deeper within the bone, compliance of the trabecular bone is essential for the joint to deform during loading and help to dissipate this energy across the layers of the joint. The different layers of the joint work in concert with one another to facilitate support and force distribution: the articular cartilage is supported by the calcified cartilage, which is supported by the subchondral bone plate, which is in turn supported by the subchondral trabecular bone and ultimately the cortical bone.

The complex balance of function between the different layers must compensate for the rate and coordinated loading of the joint, as these are the two most important factors in the bone's ability to respond to imposed stresses. Joint shape and ligamentous attachments confine joint motion and in doing so affect the pattern of response observed within the subchondral bone. The muscles or tendons which span the joint are the primary contributors of surface loads, which are generated to counteract the rotational forces secondary to the ground reaction force acting on the moment arm of the limb (2, 13) The generated forces are not equal however, as the moment arm of the tendon is typically shorter than that of the limb. Because of this, the force generated from the tendon is much larger than the ground reaction force, resulting in an amplification of contact forces within the joint and at the subchondral bone.

The joint is able to respond to repetitive loading through the adaptive processes of bone modeling and remodeling. Bone modeling is defined as bone formation and resorption at anatomically distinct sites to produce functionally and mechanically purposeful architecture (14). Bone modeling involves the geometric sculpting of bone by formation and/or resorption. Osteoclasts and osteoblasts work independently, and is typically characterized by a greater amount of bone formation than resorption. Modeling occurs at both the macroscopic and microscopic levels, with variation in the size and shape of the joints and microarchitecture observed over time. Modeling in subchondral bone typically manifests microscopically as changes in the microarchitecture, with trabecular infilling; and macroscopically as a thickening of the bone. These alterations change the mechanical properties in the bone, resulting in stiffer bone with decreased elasticity and reduced capacity for shock absorption. The bone may thicken in response to increased physical demands, as either a method to strengthen the bone or to reset the physiologic threshold, such as in cases of juvenile animals or those returning to exercise.

Bone remodeling is the coordinated activity of osteoclasts and osteoblasts to remove biomechanically inferior packets of bone and replace it with new bone (11). The processes of bone formation and resorption are asynchronously coupled, with small packets of abnormal or damaged bone resorbed by osteoclasts, followed by the recruitment of osteoblastic precursors which then differentiate and replace the removed bone (15). Importantly, there is a delay in the replacement of diseased bone, with osteoclastic processes occurring within weeks, while osteoblastic replacement of bone is slower and occurs over months. As this process occurs, the delay in new bone results in a relative osteoporosis at the site of bone replacement, as the bone is initially weakened after osteoclasts have removed the inferior bone, prior to the osteoblastic replacement with new bone. This transient osteoporosis is most notable 60–120 days after the initial injury, and clinicians must be cognizant that affected bone is at an increased risk of fracturing during this period if compounded with a complete lack of physical activity (16). For this reason, controlled activity—such as hand walking or paddock turnout—is typically recommended to mitigate the potential for fracture during this time.

**Figure 1:**
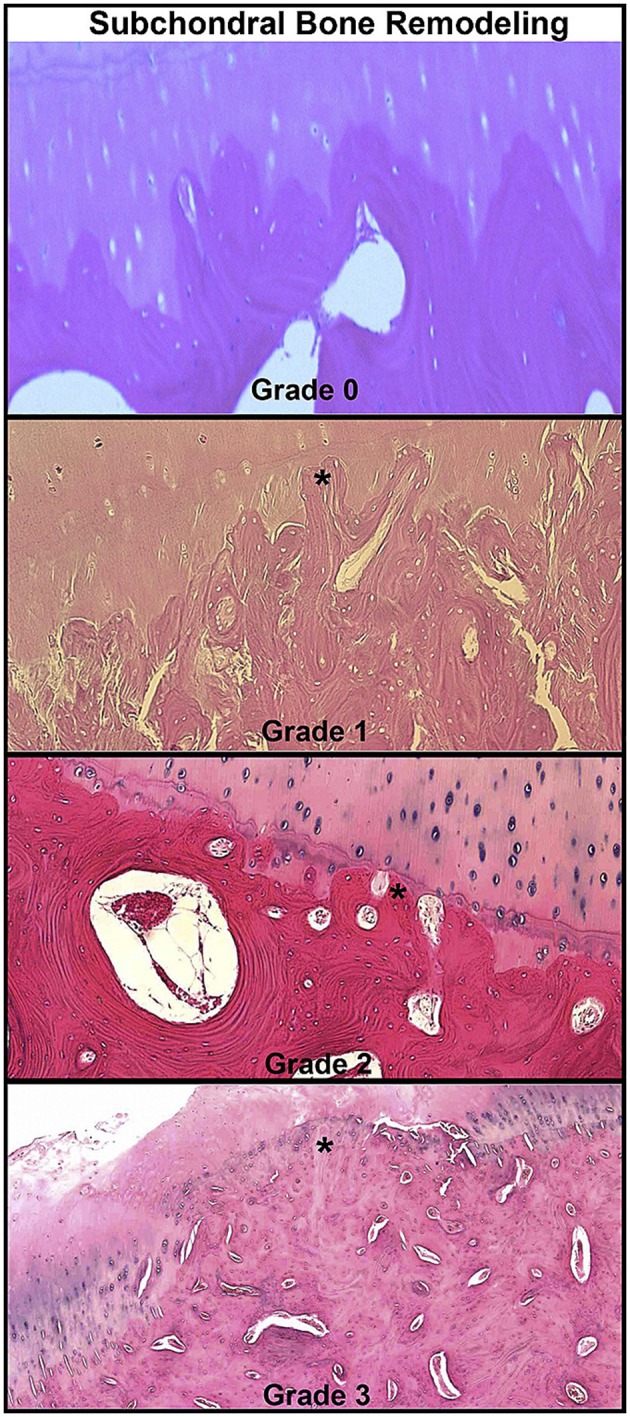
Histopathologic grading examples of osteochondral lesions in spontaneous osteoarthritis cases in equine metacarpophalangeal joints. Microscopic images showing advancement of subchondral bone remodeling through calcified cartilage layer (star). Reprinted from McIlwraith et al. (17), with permission from Elsevier.

When the adaptive capabilities of the bone are exceeded—especially in cases of corresponding degradation of the articular cartilage layer—sclerosis, osteophytes and fibrocartilaginous repair tissues are visible within the osteochondral unit. Subchondral bone follows the innate properties of all tissues, as there is a strain threshold beyond which normal adaptive processes are unable to compensate and pathological events progress resulting in subchondral bone damage. Sclerosis may be observed with subchondral bone damage, which results in a decreased elasticity within the subchondral bone plate and trabecular bone. This thickening within the subchondral bone in turn affects the ability of the articular cartilage to withstand mechanical loading, by increasing transverse stresses at the base of the articular cartilage layer, resulting in horizontal clefts within the deep zone of cartilage. With continued loading, these clefts can progress to the articular surface of the cartilage, perpetuating the cycle of OA changes within the joint (Figure 1) (18–20). The point at which this adaptive process becomes pathological is influenced by a multitude of factors, and includes (but is not limited to) location (i.e., within and between joints), horse size, speed, discipline, and amount of training (2). Clinically these changes result in the perception of pain, as the rich nerve supply of subchondral bone is one of the main mechanisms of pain perception in the joint disease (3).

The complex balance of adaptive and maladaptive bone modeling is further complicated by the fact that microdamage is observed even in well-adapted bones. Osteoclasts remove damaged bone, which is then replaced with new bone by osteoblasts. Fatigue injuries, also sometimes referred to as “repetitive stress injuries” or “chronic fatigue injuries,” have been observed most commonly in both Thoroughbred and Standardbred racehorses. These fatigue injuries occur when microdamage accumulates faster than remodeling can repair. Additionally, it is also accepted that remodeling activities are inhibited in a high load environment due to reduced recruitment of osteoclasts (16). Pathologically fatigued bone clinically manifests in one of two ways: either as overt, mechanical failure in the form of a fracture, or as biological or functional failure where subtle changes occur resulting in the inability of the bone to sustain the expected demands of activity. Biological or functional failure is typically the result of more insidious changes within the joint, resulting in abnormal or inefficient transmission of loads, incongruent articular surfaces, and may include compromised perfusion or inadequate physical support from the adjacent articular cartilage (2). Fractures of fatigued bone can be further characterized as to whether they are the result of supra-physiological loads or the result of cumulative microdamage resulting in excessive fatigue within the bone. Taken together, the more contemporary research has demonstrated strong evidence that subchondral bone changes are not simply a secondary sign of OA, but rather can be an initiating factor of degeneration of the health of the joint (21, 22).

## Subchondral bone disease and chronic fatigue injury

Historically, traumatic arthritis has been held as a condition of synovial-mediated degradation of articular cartilage and direct mechanical damage to the joint surface. More recently, the joint has been re-conceptualized as an organ system, within which multiple tissues can be damaged and contribute to overall injury and dysfunction. A general approach to understanding the fundamental mechanisms for OA have been noted as either abnormal loading on normal cartilage or normal loading on abnormal cartilage (23). Damage of the articular cartilage can occur from a variety of different circumstances, including damage to the subchondral bone, synovial membrane, fibrous joint capsule, peri-ligamentous support structures, or direct trauma to the articular cartilage itself (24). Damage to the subchondral bone has received increasing interest for its role in joint injury, as changes in the composition or mechanical properties of the subchondral bone appear to mediate some of the changes observed in OA. For example, sclerosis reduces the shock-absorbing capability of the subchondral bone, and increases the risk of shear-induced tensile failure of the articular cartilage cross-links (12); and subchondral bone has also been demonstrated as a potential source of inflammatory mediators that have been associated with degradation of deeper layers of articular cartilage (25, 26). Changes are also observed on the microarchitectural level, with the coalescing of microcracks or microfractures within the bone. Rapid, excessive bone formation may result in the development of bone sclerosis, which may be of reduced mineral quality and integrity (27). Furthermore, expansion of the trabecular bone observed in subchondral bone undergoing excessive new bone formation, reduces the size of the bone marrow spaces, potentially resulting in an ischemic state, or at the very least a change in tissue nutrition (28). Integration between the old bone and the newly added bone also takes time, and if there is a disparity between mineral properties, this may further predispose this area of bone to failure (27).

Although many terms are used by clinicians and researchers alike, the term “subchondral bone disease” most accurately represents the different phases and spectrum of pathologic changes observed within the subchondral bone. At the 2015 Dorothy Russell Havemeyer Foundation workshop in Newmarket, UK focused on subchondral bone, a consensus definition for subchondral bone disease included, “a repetitive stress injury of subchondral bone” (2). The delay between bone resorption and remodeling, where bone is resorbed at a faster rate than it is replaced, leaves the equine athlete susceptible to injury, especially when it is subjected to excessive training and stress. This type of subchondral bone injury is frequently referred to as “maladaptive,” however this term is somewhat misleading, as the bone does not truly display a maladaptive response, but rather the subchondral bone does not have the opportunity—typically due to time or ongoing physical stresses—to appropriately complete the repair processes. Compensation for these stresses is only possible up to a point, after which time the bone enters a spectrum of pathologic changes, which may be further complicated by the concurrent presence of clinical disease. The degree of the adaptive response tolerated by a given horse before showing clinical signs indicative of greater pathology appears somewhat individualized, which makes understanding the line between “normal” and “pathologic” responses difficult to distinguish.

The athletic horse is a well-studied model for repetitive stress injury, with multiple locations and manifestations of the disease. The metacarpal and metatarsal condyles are the most recognized locations of repetitive stress response and injury, with macroscopic abnormalities including increased radiopharmaceutical uptake on nuclear scintigraphy, radiolucency within the condyles on radiography, increased sclerosis within the bone on computed tomography and longitudinal fractures within the bone. Slab fractures of the carpal and tarsal bones, parasagittal fractures of the proximal phalanx, mid-body fractures of the proximal sesamoid bone, dorsoproximal fractures of the third metacarpal and metatarsal bones, wing fractures of the distal phalanx, palmar/plantar osteochondral disease, and intra-articular fragmentation are some of the other well-recognized examples of this condition. In some cases, it may be difficult to distinguish whether these injuries are primary diseases of subchondral bone, or whether they reflect more complex traumatic injuries to the bone, cartilage, and supporting soft tissues of the joint. An acute injury to a bony or ligamentous structure around or within a joint may alter weight-bearing during exercise and stimulate alterations within the subchondral bone that may result in the development of disease. Recent work in Thoroughbred racehorses has recognized a significant association between catastrophic biaxial proximal sesamoid bone fractures and disease of the subchondral bone of the third metacarpal bone of the contralateral limb (29). Subchondral bone disease is most frequently recognized in racehorses, but can affect horses in a variety of disciplines, including steeplechasers, jumpers, and three-day event horses.

**Figure 2:**
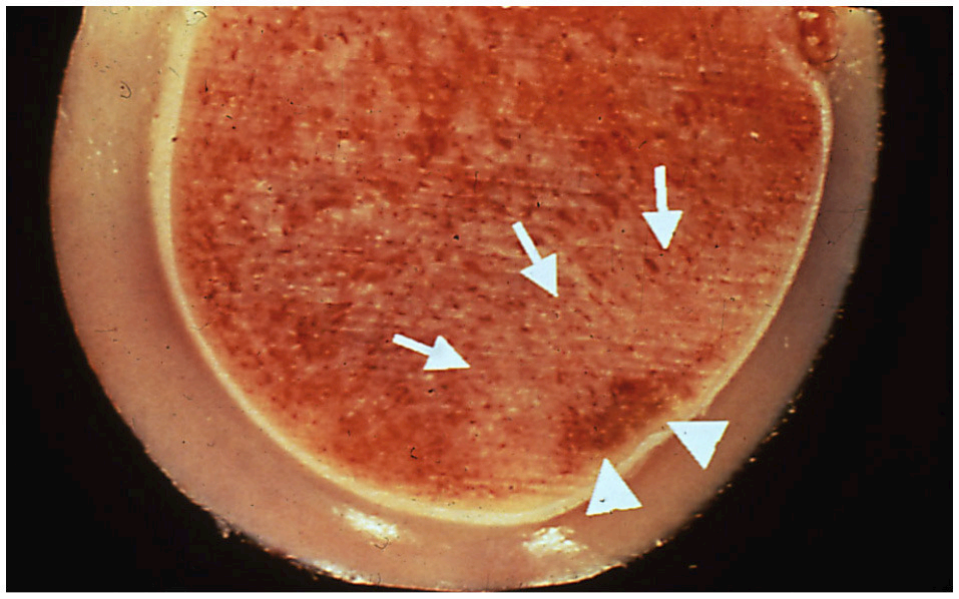
Postmortem sample of a distal metacarpus from the leg opposite to that suffering a catastrophic injury in a racehorse. Although there is intact articular cartilage, subchondral bone necrosis (arrowheads) and sclerosis (arrows) can be seen. Reprinted from McIlwraith et al. (3), with permission from Elsevier.

Investigation of injury in subchondral bone has been reported in both clinical cases and experimental models. Microdamage within the subchondral bone has been experimentally investigated using an equine model with controlled treadmill exercise, showing that it can develop early under exercising conditions (18). The metacarpal condyles of these horses displayed changes indicative of a milder version of what has been observed in clinical cases. Investigation of subchondral bone changes in clinical cases of racehorses euthanized for other catastrophic injuries has revealed changes on both a macroscopic and microscopic level. On gross examination, the metacarpo/tarsophalangeal joints from racehorses without catastrophic fractures displayed a spectrum of disease, ranging from fibrillation of the articular cartilage to focal cartilage erosions and cavitation within the subchondral bone (20). Lesions in the subchondral bone varied from thickening of the subchondral and trabecular bone to advancing sclerosis with increasing amounts of osteocyte necrosis, the presence of vascular channels filled with matrix debris and osteoclastic remodeling. Changes in the subchondral bone were not limited to architectural microdamage alone, as osteocyte death was also identified. Changes in the subchondral bone—such as necrosis and sclerosis—could also be present in the face of intact articular cartilage (as has been observed in the palmar/plantar aspect of the distal third metacarpal/tarsal bones) (20). In many of these cases, on gross examination the articular cartilage appeared largely viable, with limited erosion and degeneration within the superficial layers (Figure 2). Focal disruption of the calcified cartilage layer appeared to result in cartilage in-folding. The disparity between these experimental and clinical findings is not necessarily unexpected, as it is unrealistic to believe clinical conditions can be perfectly modeled in a laboratory setting. Taken together however, these findings would suggest that microdamage within the subchondral bone not only results in the loss of mechanical support to the articular cartilage, but local factors (i.e., cytokines) released from the bone can influence—potentially permanently—the state and health of the articular cartilage (11, 18, 20). Furthermore, progressive injury within the subchondral bone can result in complete failure, with pathologic changes within the third metacarpal/tarsal bones culminating in condylar fracture (11, 30–32). More recently, the term “impact fracture” has been used to describe these types of pathologic fractures that correspond to areas of radiographic lucency within the bone (33).

Chronic fatigue injury has also been used to describe injury to the subchondral bone. Chronic fatigue injury is a broader and more encompassing term than “subchondral bone disease,” as the subchondral bone is typically not the only tissue affected. Chronic fatigue injuries result from cyclic loading of the tissue below the biomechanical threshold of tissue failure, and occurs commonly in the subchondral bone of the equine athlete (3, 11). Currently, it seems that chronic fatigue injuries and repetitive stress injuries are terms used interchangeably throughout the literature and amongst clinicians, suggesting these terms are frequently used to describe the same underlying disease processes of subchondral bone. As more is understood about the biological behavior of subchondral bone under abnormal physiologic conditions, further clarification or consensus about these terms will likely be important. Chronic fatigue injury in the subchondral bone was initially described in clinical cases of injured racehorses (34); and validated through numerous clinical studies and experimental models (18–20, 35–37). There are three mechanisms by which damage can occur: (1) microdamage formation within the tissues; (2) an area of weakness within tissues secondary to biomechanical and tissue responses to cyclic loading, predisposing this tissue to damage; or (3) adaptive tissue responses that chronically fatigue the tissue, resulting in a change in material properties and ultimately to injury (3). One of the best clinical examples of chronic fatigue injury is in the palmar aspect of the third metacarpal condyles. In young Thoroughbred racehorses in training, sclerosis in the palmar aspect of the condyles is common, but the difficulty lies in discerning when these changes are indicative of a pathologic response. It is rare to observe clinical abnormalities in this region until these processes have resulted in subchondral bone pain or gross damage within the joint (38).

Advances in diagnostic imaging have improved identification of these pathologic changes and is an attractive avenue for further research into understanding subchondral bone disease. Although the significance of osteochondral injury in joint disease has been well-discussed (39), our understanding of the complex relationship between the subchondral bone and articular cartilage in repetitive stress injury and chronic fatigue injury continues to evolve, as great progress in understanding have and will likely continue to be made over the next decade through advancements in imaging and experimental disease models.

## Principles of diagnosis and diagnostic imaging

The tenets of diagnosis for subchondral bone disease remain the same as for many other musculoskeletal conditions in the horse—a thorough clinical examination (including static and dynamic, and subjective and objective evaluations) to localize the source of lameness, diagnostic analgesia, and diagnostic imaging examination. Subchondral bone injury may be identified on standard radiographic projections, but also may be missed depending on the location and time-course of the disease. Diseased bone may radiographically have the appearance of areas of decreased opacity surrounded by areas of increased opacity, or may be visible as a distinct fracture. A lack of radiographically observable abnormalities does not rule out the presence of subchondral bone disease and repeat imaging (in 10–14 days) or use of a different, more advanced imaging modality should be considered. Depending on the severity and chronicity of the injury, nuclear scintigraphy, magnetic resonance imaging (MRI), and computed tomography (CT) can be considered for further evaluation of changes within the subchondral bone. In severe cases where overlying articular cartilage damage is also present, diagnostic arthroscopy may be considered for further evaluation of the subchondral bone. Volumetric imaging techniques, such as MRI and CT that provide information in three-dimensions have arguably revolutionized our clinical ability to assess subchondral bone and the health of the joint as a whole.

**Figure 3:**
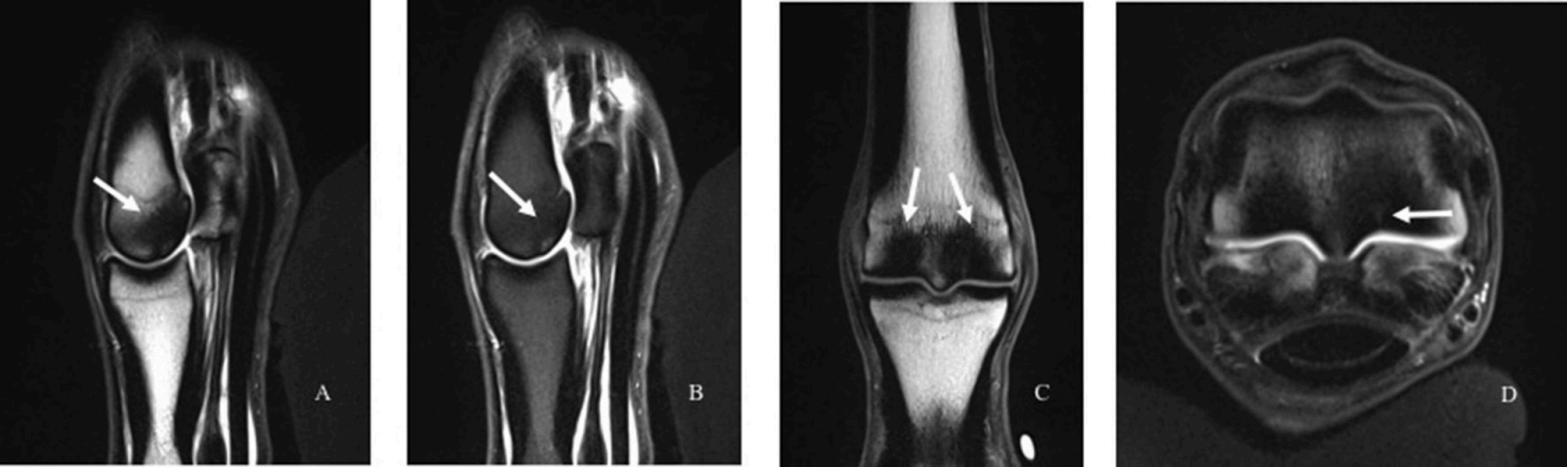
Images of subchondral bone injury as detected using magnetic resonance imaging (MRI). An intermediate-weighted T1 TSE sequence with **(A)** and without **(B)** fat suppression in the sagittal **(A, B)**, dorsal **(C)**, and transverse **(D)** planes. There is marked subchondral and trabecular bone sclerosis in the palmar aspect of the third metacarpal condyle (white arrows), with the lateral condyle slightly more affected than the medial condyle. There is a fissure visible within the bone on the palmar-axial aspect of the lateral condyle that also exhibits increased signal (**D**, arrow).

Magnetic resonance imaging has facilitated the identification of diseased subchondral bone. Terms such as "bone marrow edema,” “bone bruise,” or “bone contusion” have all been used to describe what are now referred to as bone marrow lesions (40). These terms all describe lesions within the subchondral bone with high signal intensity on fluid-sensitive sequences (Figure 3). Bone marrow lesions have a characteristic decreased signal intensity on T1-weighted images and increased signal intensity of T2-weighted images (41), Multiple theories and etiologies have been proposed for the formation of bone marrow lesions, and histologic examination of these lesions have identified a wide spectrum of abnormalities (42). More recently, compelling reports have been published citing bone marrow lesions as early indicators of structural deterioration of the joint and may serve as a marker for maladaptive changes within the subchondral bone and articular cartilage (43–46). Further research is essential and forthcoming in this field with the continued development of high-field MRI systems that can provide increased detail.

In addition to identification of bone marrow lesions, increased bone mineral density or sclerosis is frequently identified on MRI in areas of signal changes. These changes are identified as areas of low signal intensity, but this depends on the sequences used for evaluation. Furthermore, diseased subchondral bone is not always sclerotic and although increased bone density may be present, signal changes on MRI are non-specific and may represent an increased volume of lower density bone. Increased signal intensity on MRI should be interpreted with caution, as this may not truly represent the trabecular thickening inherent to sclerosis (2).

**Figure 4:**
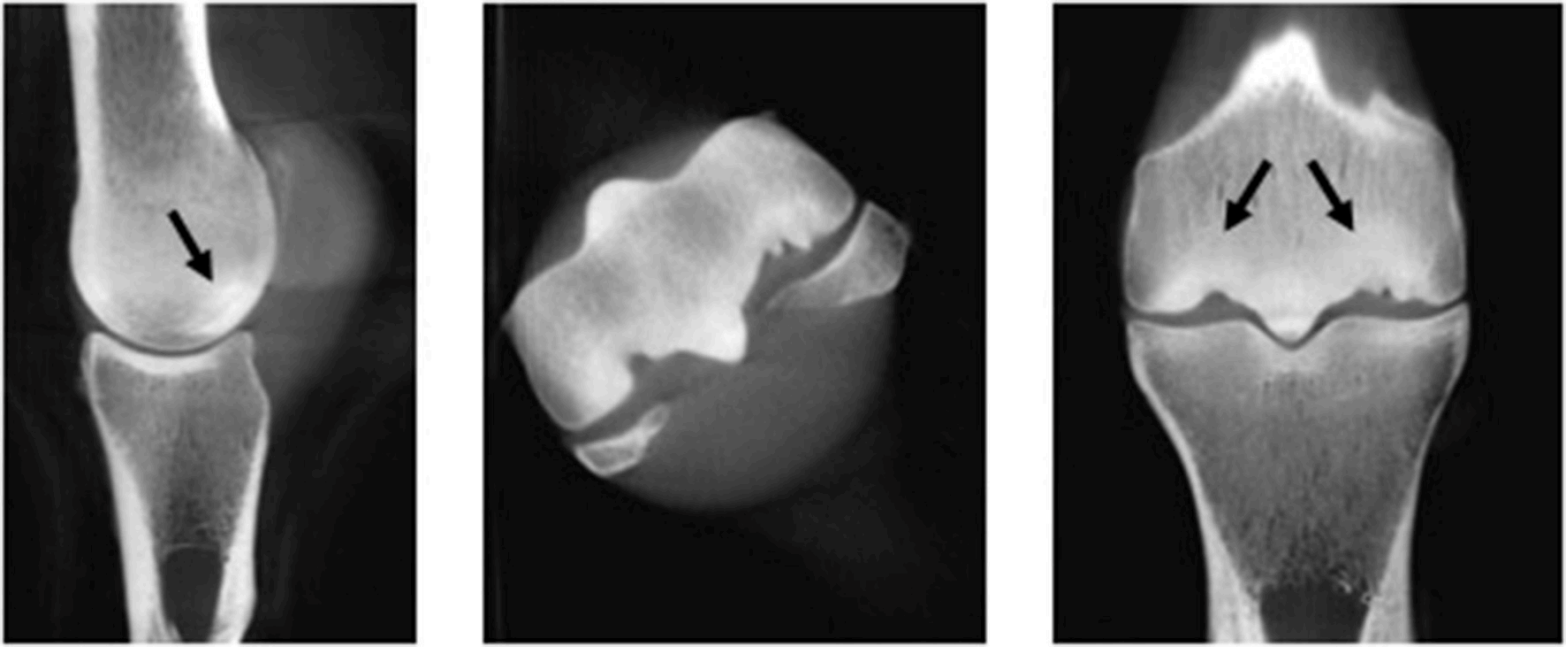
Image of the distal metacarpus of a 10-year-old Thoroughbred racehorse obtained post-mortem with marked palmar osteochondral disease obtained using computed tomography (CT) in the sagittal, transverse and dorsal planes. Subchondral bone sclerosis and lysis (black arrows) and extensive articular cartilage loss is visible on these images and was present on gross evaluation.

Magnetic resonance imaging remains as the only modality capable of being used to identify fluid (e.g., hemorrhage, edema, fibrosis, necrosis) within bone; however CT has superior resolution for structural imaging of bone. Fine bone detail can be very challenging to identify using MRI, as the appearance of bone and soft tissue can overlap. In cases of confluent tissue—such as in a joint with osseous proliferation with adjacent soft tissue thickening—the signal intensity of MRI will be the same and it can be challenging or impossible to distinguish between the structures. The volumetric information from reconstructed CT images can illustrate subtle to extensive internal and external osseous remodeling (Figure 4). In specific sites, remodeling changes observed on CT have been validated to indicate pathologic change and impending fractures (47). The tissue density observed on CT can be translated into numerical values known as Hounsfield units (HU). Information about the specific densities of the bone may provide valuable insight into unique patterns of bone change, and furthermore provides an objective metric for comparison if serial examinations are performed.

The information afforded through volumetric imaging modalities such as CT and MRI, have increased our knowledge in the behavior of subchondral bone and its response to training and injury. Historically, CT and MRI evaluation in the equine patient has been limited due to gantry size or requirements for general anesthesia. Recent developments have yielded MR systems and rapid-scan CT units dedicated for equine use with an enlarged gantry size (Pegaso, Epica Medical Innovations, San Clemente, CA USA) or separate generator-receiver robotically-controlled arms (4DDI Equimagine, Equine Imaging LLC, Milwaukee, WI, USA), all of which can be used in the standing horse. MRI and CT should be considered as complimentary to one another when evaluating the subchondral bone, as each provides unique and valuable information. An additional notable volumetric imaging modality that is receiving increasing attention as a developing technology for assessment of subchondral bone is positron emission computed tomography (PET). Recent work has demonstrated that PET is able to identify lesions that were not visible using other imaging modalities, and furthermore to distinguish between active and inactive lesions (48). As PET gains further justification, the potential applications for evaluation of subchondral bone injury will likely warrant further discussion. With increasing accessibility to these volumetric imaging modalities and evolving understanding of the role of subchondral bone in joint disease, it is likely that assessment of subchondral bone will become an integral part of diagnostic imaging for veterinary patients affected by orthopedic conditions.

## Treatment and prevention

The causes of pain associated with diseased subchondral bone, including bone resorption, still remain poorly understood. Proposed contributors to pain may include instability, increased intraosseous pressure, hypertension, and impingement of sensitive structures such as the periosteum, ligaments or joint capsule. The two consensus goals for treatment of subchondral bone disease include: (1) restoring function and (2) preventing progression of disease to failure through pain relief and restoration of normal bone architecture (2). It is challenging to create a specific treatment regimen since subchondral bone disease encompasses a large spectrum of abnormalities. The plethora of methods proposed to treat subchondral bone disease reflects the variety of challenges associated with managing this condition. If pain or lameness are recognized early in the maladaptive response process, exercise modifications including altering the intensity, duration or type (e.g., reduced work, turn out, swimming, underwater treadmilling) for a short period may be all that is necessary to resolve clinical signs. A notable critique of these alternative training methods is that they utilize instability, or alterations in the distribution of weightbearing forces across the articular surfaces (49). Instability has been shown to prevent vessel ingrowth, and despite the fact that subchondral bone disease is not necessarily a primary vascular condition, avascular areas are commonly present within diseased subchondral bone (2). Bisphosphonates have also been used commonly to treat subchondral bone disease, but their potential effectiveness has yet to be fully elucidated. Proponents of bisphosphonate therapy argue that inhibition of osteoclastic activity benefits those cases undergoing active degradation of the subchondral bone, and additional analgesic and anti-inflammatory effects have also been suggested (2, 50, 51). Despite these potential benefits and positive reports in the human medical literature, there has been no definitive consensus in the treatment of subchondral bone disease using bisphosphonates in veterinary species. Bisphosphonates are also not licensed for use in juvenile horses, which likely represent the largest population of cases of subchondral bone injury through the racehorse industry.

The frequency and intensity that subchondral bone disease is observed would justify this as an economic issue, however there is a paucity of data on the incidence and specific demographics of this disease. The inherent difficulties in identifying subchondral bone disease prior to the occurrence of more severe sequelae makes epidemiologic studies very challenging. At the current time, the best prevention of subchondral bone disease is focused on reducing risk. Despite inconsistent agreement in a specific definition for clinical signs of subchondral bone disease, it is well-agreed that cumulative exercise is associated with an increased risk of subchondral bone disease (2). Exercise and training regimes should be tailored to each specific horse, with specific attention to the clinical condition and response of the horse. Published work has recognized that exposure to exercise at the end of growth, but before skeletal maturity is beneficial for subchondral bone development, and this must be balanced with the adaptive response of each animal. Further work is essential in order to understand those cases that may be at an increased risk for development of subchondral bone disease through the use of multi-modality imaging or potentially biomarker panels. Despite the voids in the current knowledge about subchondral bone disease, exercise modulation will likely remain a central tenet of disease management.

## Conclusion

Substantial insight has been gained about the biomechanical influences of the joint on the subchondral bone, with the relationship between subchondral bone injury and articular cartilage loss and the development of degenerative joint disease only beginning to be elucidated. Continued investigation of the adaptive and maladaptive changes within the subchondral bone by researchers and clinicians alike will continue to yield valuable information about the behavior of this unique component of the joint. Further discovery of the delicate balance of factors that maintain the integrity of the subchondral bone and homeostasis within the joint will surely enhance and direct our understanding of subchondral bone disease in across both veterinary and human patients.

## Author contributions

Both HS and CK contributed equally to the preparation, developing, writing and editing of the review article submitted.

### Conflict of interest statement

The authors declare that the research was conducted in the absence of any commercial or financial relationships that could be construed as a potential conflict of interest.

## References

[1] Gomoll AH Farr J editors. The osteochondral unit. In: Cartilage Restoration. New York, NY: Springer New York (2014). p. 9–15.

[2] Smith MRW Kawcak CE McIlwraith CW. Science in brief: report on the Havemeyer Foundation workshop on subchondral bone problems in the equine athlete. Equine Vet J. (2016) 48:6–8. 10.1111/evj.12518 26663405

[3] McIlwraith CW Frisbie DD Kawcak CE van Weeren R Joint Disease in the Horse. 2nd ed. St. Louis, MO: Elsevier Inc (2016).

[4] Clark JM Huber JD. The structure of the human subchondral plate. J Bone Joint Surg Br. (1990) 72:866–73. 10.1302/0301-620X.72B5.2211774 2211774

[5] Radin EL Paul IL. Response of joints to impact loading. I. in vitro wear. Arthritis Rheum. (1971) 14:356–62. 10.1002/art.1780140306 5562019

[6] Simon SR Radin EL Paul IL Rose RM. The response of joints to impact loading — II *In vivo* behavior of subchondral bone. J Biomech. (1972) 5:267–72. 10.1016/0021-9290(72)90042-5 4269623

[7] Radin EL Parker HG Pugh JW Steinberg RS Paul IL Rose RM Response of joints to impact loading — III: Relationship between trabecular microfractures and cartilage degeneration. J Biomech. (1973) 6:51–7. 10.1016/0021-9290(73)90037-7 4693868

[8] Brama PA Holopainen J van Weeren PR Firth EC Helminen HJ Hyttinen MM. Influence of exercise and joint topography on depth-related spatial distribution of proteoglycan and collagen content in immature equine articular cartilage. Equine Vet J. (2009) 41:557–63. 10.2746/042516409X424162 19803051

[9] Mankin H Radin E Structure and function of joints. In: McCarthy D editors. Arthritis and Allied Conditions: A Textbook of Rheumatology. 12th Ed. Philadelphia, PA: Lea & Febiger (1993). p. 189.

[10] Auer JA Stick JA editors. Equine Surgery. 4th Ed. St. Louis, MO (2012).

[11] Kawcak CE McIlwraith CW Norrdin RW Park RD James SP. The role of subchondral bone in joint disease: a review. Equine Vet J. (2001) 33:120–6. 10.1111/j.2042-3306.2001.tb00589.x 11266060

[12] Radin EL Rose RM. Role of subchondral bone in the initiation and progression of cartilage damage. Clin Orthop Relat Res. (1986) 34–40. 10.1097/00003086-198612000-00005 3780104

[13] Merritt JS Davies HMS Burvill C Pandy MG Calculation of joint reaction forces in the equine distal forelimb during walking and trotting. Proc Front Converg Biosci Inf Technol. (2007) 2008:587–90. 10.1109/FBIT.2007.152

[14] Frost HM. Skeletal structural adaptations to mechanical usage (SATMU): 1. Redefining Wolff's Law: the bone modeling problem. Anat Rec. (1990) 226:403–13. 2184695 10.1002/ar.1092260402

[15] Sims NA Martin TJ. Coupling the activities of bone formation and resorption: a multitude of signals within the basic multicellular unit. Bonekey Rep. (2014) 3:481. 10.1038/bonekey.2013.215 24466412 PMC3899560

[16] Holmes JM Mirams M Mackie EJ Whitton RC. Thoroughbred horses in race training have lower levels of subchondral bone remodelling in highly loaded regions of the distal metacarpus compared to horses resting from training. Vet J. (2014) 202:443–7. 10.1016/j.tvjl.2014.09.010 25296852

[17] McIlwraith CW Frisbie DD Kawcak CE Fuller CJ Hurtig M Cruz A. The OARSI histopathology initiative – recommendations for histological assessments of osteoarthritis in the horse. Osteoarthr Cartil. (2010) 18(Suppl. 3):S93–105. 10.1016/j.joca.2010.05.031 20864027

[18] Kawcak CE McIlwraith CW Norrdin RW Park RD Steyn PS. Clinical effects of exercise on subchondral bone of carpal and metacarpophalangeal joints in horses. Am J Vet Res. (2000) 61:1252–8. 10.2460/ajvr.2000.61.1252 11039557

[19] Norrdin RW Kawcak CE Capwell BA McIlwraith CW. Calcified cartilage morphometry and its relation to subchondral bone remodeling in equine arthrosis. Bone (1999) 24:109–14. 10.1016/S8756-3282(98)00157-4 9951778

[20] Norrdin RW Kawcak CE Capwell BA McIlwraith CW. Subchondral bone failure in an equine model of overload arthrosis. Bone (1998) 22:133–9. 10.1016/S8756-3282(97)00253-6 9477236

[21] Lajeunesse D Massicotte F Pelletier JP Martel-Pelletier J. Subchondral bone sclerosis in osteoarthritis: not just an innocent bystander. Mod Rheumatol. (2003) 13:7–14. 10.3109/s101650300001 24387110

[22] Radin E. Subchondral bone changes and cartilage damage. Equine Vet J. (1999) 31:94–5. 10.1111/j.2042-3306.1999.tb03799.x 10213419

[23] Goldring MB Goldring SR. Osteoarthritis. J Cell Physiol. (2007) 213:626–34. 10.1002/jcp.21258 17786965

[24] Mcilwraith CW From arthroscopy to gene therapy–30 years of looking in joints. In: AAEP Proceedings (2005). p. 65–113.

[25] Berenbaum F Osteoarthritis as an inflammatory disease (osteoarthritis is not osteoarthrosis!). Osteoarthr Cartil. (2013) 21:16–21. 10.1016/j.joca.2012.11.012 23194896

[26] Kapoor M Martel-Pelletier J Lajeunesse D Pelletier JP Fahmi H. Role of proinflammatory cytokines in the pathophysiology of osteoarthritis. Nat Rev Rheumatol. (2011) 7:33–42. 10.1038/nrrheum.2010.196 21119608

[27] Boyde A. The real response of bone to exercise. J Anat. (2003) 203:173–89. 10.1046/j.1469-7580.2003.00213.x 12924818 PMC1571152

[28] Pool R Pathological manifestations of joint disease in the athletic horse. In: Mcilwraith CW Trotter GW editors. Joint Disease in the Horse. 1st Ed. Philadelphia, PA: Saunders Elsevier (1996). p. 98–9.

[29] Peloso JG Vogler JB Cohen ND Marquis P Hilt L. Association of catastrophic biaxial fracture of the proximal sesamoid bones with bony changes of the metacarpophalangeal joint identified by standing magnetic resonance imaging in cadaveric forelimbs of Thoroughbred racehorses. J Am Vet Med Assoc. (2015) 246:661–73. 10.2460/javma.246.6.661 25719849

[30] Riggs C. Aetiopathogenesis of parasagittal fractures of the distal condyles of the third metacarpal and third metatarsal bones - review of the literature. Equine Vet J. (1999) 31:116–20. 10.1111/j.2042-3306.1999.tb03803.x 10213423

[31] Riggs C Whitehouse GH Boyde A. Structural variation of the distal condyles of the third metacarpal and third metatarsal bones in the horse. Equine Vet J. (1999) 31:130–9. 10.1111/j.2042-3306.1999.tb03806.x 10213425

[32] Martinelli M Subchondral bone and injury. Equine Vet Educ. (2009) 21:253–6. 10.2746/095777309X431311

[33] Cullimore A Finnie J Marmion W Booth TM Severe lameness associated with an impact fracture of the proximal phalanx in a filly. Equine Vet Educ. (2009) 21:247–51. 10.2746/095777309X409901

[34] Pool RR Meagher DM. Pathologic Findings and Pathogenesis of Racetrack Injuries. Vet Clin North Am Equine Pract. (1990) 6:1–30. 10.1016/S0749-0739(17)30555-2 2187565

[35] Martig S Chen W Lee PV Whitton RC. Bone fatigue and its implications for injuries in racehorses. Equine Vet J. (2014) 46:408–15. 10.1111/evj.12241 24528139

[36] Drum MG Les CM Park RD McIlwraith CW Kawcak CE. Comparison of mean bone densities of three preparations of the distal portion of the equine third metacarpal bone measured by use of quantitative computed tomography. Am J Vet Res. (2008) 69:891–3. 10.2460/ajvr.69.7.891 18593241

[37] Turley SM Thambyah A Riggs CM Firth EC Broom ND. Microstructural changes in cartilage and bone related to repetitive overloading in an equine athlete model. J Anat. (2014) 224:647–58. 10.1111/joa.12177 24689513 PMC4025892

[38] Tull TM Bramlage LR. Racing prognosis after cumulative stress-induced injury of the distal portion of the third metacarpal and third metatarsal bones in Thoroughbred racehorses: 55 cases (2000–2009). J Am Vet Med Assoc. (2011) 238:1316–22. 10.2460/javma.238.10.1316 21568778

[39] Riggs CM Osteochondral injury and joint disease in the athletic horse. Equine Vet Educ. (2010) 18:100–12. 10.1111/j.2042-3292.2006.tb00426.x

[40] Eriksen EF. Treatment of bone marrow lesions (bone marrow edema). Bonekey Rep. (2015) 4:755. 10.1038/bonekey.2015.124 26644910 PMC4662576

[41] Bonadio MB Filho AGO Helito CP Stump XM Demange MK. Bone Marrow Lesion: Image, Clinical Presentation, and Treatment. Magn Reson Insights (2017) 10:1178623X17703382. 10.1177/1178623x17703382 28579795 PMC5428162

[42] Zanetti M Bruder E Romero J Hodler J. Bone marrow edema pattern in osteoarthritic knees: correlation between MR imaging and histologic findings. Radiology (2000) 215:835–40. 10.1148/radiology.215.3.r00jn05835 10831707

[43] Felson DT McLaughlin S Goggins J LaValley MP Gale ME Totterman S. Bone marrow edema and its relation to progression of knee osteoarthritis. Ann Intern Med. (2003) 139:330. 10.7326/0003-4819-139-5_Part_1-200309020-00008 12965941

[44] Xu L Hayashi D Roemer FW Felson DT Guermazi A. Magnetic resonance imaging of subchondral bone marrow lesions in association with osteoarthritis. Semin Arthritis Rheum. (2012) 42:105–18. 10.1016/j.semarthrit.2012.03.009 22542276 PMC3653632

[45] Hunter DJ Zhang Y Niu J Goggins J Amin S LaValley MP. Increase in bone marrow lesions associated with cartilage loss: A longitudinal magnetic resonance imaging study of knee osteoarthritis. Arthritis Rheum. (2006) 54:1529–35. 10.1002/art.21789 16646037

[46] Haavardsholm EA Bøyesen P Østergaard M Schildvold A Kvien TK. Magnetic resonance imaging findings in 84 patients with early rheumatoid arthritis: bone marrow oedema predicts erosive progression. Ann Rheum Dis. (2008) 67:794–800. 10.1136/ard.2007.071977 17981915

[47] Tucker RL Sande RD. Computed Tomography and Magnetic Resonance Imaging in Equine Musculoskeletal Conditions. Vet Clin North Am Equine Pract. (2001) 17:145–57. 10.1016/S0749-0739(17)30080-9 11488041

[48] Spriet M Espinosa P Kyme AZ Stepanov P Zavarzin V Schaeffer S. Positron emission tomography of the equine distal limb: exploratory study. Vet Radiol Ultrasound. (2016) 57:630–8. 10.1111/vru.12430 27699910

[49] King MR Haussler KK Kawcak CE McIlwraith CW Reiser RF Mechanisms of aquatic therapy and its potential use in managing equine osteoarthritis. Equine Vet Educ. (2013) 25:204–9. 10.1111/j.2042-3292.2012.00389.x

[50] Bonabello A Galmozzi M, Bruzzese T, Zara GP. Analgesic effect of bisphosphonates in mice. Pain (2001) 91:269–75. 10.1016/S0304-3959(00)00447-4 11275384

[51] Maksymowych WP Bisphosphonates, anti-inflammatory properties. Curr Med Chem Anti- Inflamm Anti-Allergy Agents (2002) 1:15–28. 10.2174/1568014024606539

